# What is the role of locoregional anesthesia in breast surgery? A systematic literature review focused on pain intensity, opioid consumption, adverse events, and patient satisfaction

**DOI:** 10.1186/s12871-020-01206-4

**Published:** 2020-11-23

**Authors:** Pasquale Sansone, Luca Gregorio Giaccari, Mario Faenza, Pasquale Di Costanzo, Sara Izzo, Caterina Aurilio, Francesco Coppolino, Maria Beatrice Passavanti, Vincenzo Pota, Maria Caterina Pace

**Affiliations:** 1 Department of Woman, Child and General and Specialized Surgery, University of Campania “Luigi Vanvitelli”, Piazza Luigi Miraglia, 2, Naples, Italy; 2 Multidisciplinary Department of Medical Surgical and Dental Sciences – Plastic Surgery Unit, University of Campania “Luigi Vanvitelli”, Naples, Italy

**Keywords:** Breast surgery, Mastectomy, Locoregional anesthesia, Pain intensity, Opioid consumption, Adverse events, Patient satisfaction

## Abstract

**Background:**

Breast surgery in the United States is common. Pain affects up to 50% of women undergoing breast surgery and can interfere with postoperative outcomes. General anesthesia is the conventional, most frequently used anaesthetic technique. Various locoregional anesthetic techniques are also used for breast surgeries. A systematic review of the use of locoregional anesthesia for postoperative pain in breast surgery is needed to clarify its role in pain management.

**Objectives:**

To systematically review literature to establish the efficacy and the safety of locoregional anesthesia used in the treatment of pain after breast surgery.

**Methods:**

Embase, MEDLINE, Google Scholar and Cochrane Central Trials Register were systematically searched in Mars 2020 for studies examining locoregional anesthesia for management of pain in adults after breast surgery. The methodological quality of the studies and their results were appraised using the Consensus-based Standards for the Selection of Health Measurement Instruments (COSMIN) checklist and specific measurement properties criteria, respectively.

**Results:**

Nineteen studies evaluating locoregional anesthesia were included: 1058 patients underwent lumpectomy/mastectomy, 142 breast augmentation and 79 breast reduction. Locoregional anesthesia provides effective anesthesia and analgesia in the perioperative setting, however no statistically significant difference emerged if compared to other techniques. For mastectomy only, the use of locoregional techniques reduces pain in the first hour after the end of the surgery if compared to other procedures (*p* = 0.02). Other potentially beneficial effects of locoregional anesthesia include decreased need for opioids, decreased postoperative nausea and vomiting, fewer complications and increased patient satisfaction. All this improves postoperative recovery and shortens hospitalization stay. In none of these cases, locoregional anesthesia was statistically superior to other techniques.

**Conclusion:**

The results of our review showed no differences between locoregional anesthesia and other techniques in the management of breast surgery. Locoregional techniques are superior in reducing pain in the first hour after mastectomy.

## Background

### Rationale

Breast surgery in the United States is common. In 2020, an estimated 276,480 new cases of invasive breast cancer will be diagnosed among women and approximately 80% of patients will have surgery to remove their primary tumour [1]. In addition, an increasing number of women are turning to plastic surgeons for interventions of cosmetic: among these breast augmentation is the first procedure performed in the United States [2].

Pain affects up to 50% of women undergoing breast surgery and can interfere with postoperative outcomes. Breast pain is one of the factors determining patient distress, long hospital stay, and an increase in post-surgical admissions to the hospital [3].

Nociceptive/inflammatory pain is caused by tissue damage, whereas neuropathic pain is the consequence of a central and peripheral nerve damage [4], especially the intercostal nerves from T2 to T6. Neuropathic pain typically begins immediately after breast surgery and can be persistent, sometimes even for months [3].

The pain control is the main objective of anesthesia in breast surgery. The correct management of acute postoperative pain is essential to improve patient outcome and satisfaction.

Various anesthetic agents, devices, and strategies are currently available. For a long time, intravenous analgesia has been the main technique for postoperative pain relief. Over the years, the growing number of surgical procedures for breast cancer and cosmetic treatment has however stimulated the development of new anesthetic techniques with improved pain reduction and safety, and fewer complications.

The international guidelines recommended the use of a multimodal analgesia [5, 6]. Regional anesthesia techniques are effective as a component of multimodal analgesia for management of postoperative pain associated with a number of surgical procedures. These techniques can be administered as a single shot or a continuous catheter, both prior to surgical incision or after surgery [5, 6]. Local anesthetics infiltration also shows benefit for the surgical procedure. Wound infiltration can be performed either as a single injection of local anesthetic typically at the conclusion of surgery or as a continuous infusion of local anesthetic through a catheter at the incision site prior to skin closure [5, 6]. Finally, the international guidelines suggest the use of intravenous (IV) lidocaine, especially in patients underwent open or laparoscopic abdominal surgical procedures [5, 6]. Perioperative lidocaine infusion may be considered for patients undergoing mastectomy [7].

### Objectives

We undertook this systematic review to identify the potential clinical role of locoregional anesthesia for breast surgery. We aimed to establish the efficacy and the safety of locoregional anesthesia in the pain management after breast surgery. Postoperative pain severity and opioid consumption during the first 48 h were designated as co-primary outcomes. For secondary objectives, we aimed to examine the effects on the immediate quality of recovery, in relation to adverse effects and patient satisfaction.

## Methods

### Protocol and registration

We performed a systematic review based on Preferred Reporting Items for Systematic Reviews and Meta-Analyses (PRISMA) statement [8]. Randomized controlled trials that compared the effects of locoregional anesthesia to systemic analgesia alone in patients undergoing breast surgery were sought. Studies were evaluated using a pre-designed protocol. The protocol was not published, and the review was not registered with the International prospective register of systematic reviews (PROSPERO).

### Eligibility criteria

The population, intervention, comparison, and outcome (PICO) criteria were applied to the research question (see Table 1). Patients older than 18 years undergoing breast surgery were considered as the population (P); the intervention (I) was postoperative analgesia with locoregional anesthesia techniques; the comparison (C) concept was standard pain treatment provided in each study; pain intensity, opioid consumption, adverse events (AEs), and patient satisfaction were considered the outcomes (O) for this systematic review. We included randomized controlled trials (RCTs) published from 2010 to the present. No language restrictions were placed on study inclusion.

**Table 1:** PICO criteria for including studies

**Population**	Patients of at least 18 years undergoing breast surgery.
**Intervetion**	Postoperative analgesia with locoregional anesthesia techniques.
**Comparator**	Standard pain treatment.
**Outcomes**	Pain Intensity, Opioid Consumption, Adverse Events, Patient Satisfaction.
**Study TYPE**	Randomized Controlled Trial.
**Time**	From 2010 to present.

### Literature search

We identified the articles by searching electronic databases (Embase, MEDLINE, Google Scholar and Cochrane Central Trials Register). We included other relevant studies from the reference lists of systematic reviews and meta-analyses. These databases were initially searched from Mars 2020.

The search strategy was developed using medical subject headings and keywords relating to the central research question of this paper. Namely, the search terms included in the search strategy covered the following key domains: *“breast augmentation”, “breast reduction”, “mastectomy”, “mastopexy”, “local anesthetic agent”, “postoperative pain”* and *“randomized clinical trial”.*

We applied no language restrictions in searches.

The studies included in this review evaluated adult patients undergoing breast surgery and receiving any type of locoregional anesthesia to treat postoperative pain.

### Primary outcomes

Pain scores and opioid consumption in the first 48 h postoperatively were the primary outcomes of interest. Pain intensity was assessed via a Numeric Rating Scale (NRS) at 1, 6, 12, 24 and 48 h after surgery. Pain intensity data assessed by means other than a zero to 10 NRS were converted to this scale. The other primary outcome was the average per patient opioid consumption in the Post-Anesthesia Care Unit (PACU) and in the 48 h after surgery. Opioid consumption was converted to morphine sulfate equivalents [9].

### Secondary outcomes

We extracted data on the following secondary outcomes:

1. Adverse Events (AEs) were recorded. Evaluated safety outcomes included postoperative opioid related side-effects (postoperative nausea and vomiting, sedation/respiratory depression, pruritus, hypotension, urinary retention, or constipation), and complications related to the nerve block (pneumothorax, block failure, or local anesthetic systemic toxicity). Complications during wound healing were also analysed.
2. Patient Satisfaction. All measures of patient satisfaction were reported as “satisfied” and “not satisfied”.

### Selection of studies

Two independent reviewers (P.S. and L.G.G.) initially assessed the results from the literature search based on title and abstract. The full-text citations of potentially eligible articles were subsequently retrieved and reviewed again by the same two independent reviewers. In case of disagreement between the two reviewers on eligibility, a discussion was initiated. If consensus could not be reached after discussion, a third reviewer (M.C.P.) evaluated the study and made the final decision.

The methodological quality of the included studies was evaluated and rated using the COSMIN checklist, based on a 4-point rating scale.

### Data extraction and management

A standardized data extraction form was used. Data collected included information relating to:

- Age, weight, height and body mass index (BMI) of participants;
- Number of participants enrolled and completing the study;
- Type of operation;
- Regional anesthesia technique (local anesthetic and dose);
- Pain intensity for all-time points at which it was measured;
- Opioid consumption;
- Patient satisfaction;
- Severity or incidence of adverse events.

### Statistical analyses

For continuous outcomes, we extracted the mean and standard deviation (SD). In situations where these are not reported, the median and interquartile range were used to approximate these values. Similarly, in situations where the mean and 95% CI are reported, statistical conversions were used to estimate the mean and SD.

For dichotomous outcomes (side effects, complications), data were converted to overall incidence numbers.

We designated a *p* value < 0.05 as a threshold of statistical significance for the primary and secondary outcomes. All tests were two-tailed.

## Results

**Fig. 1:**
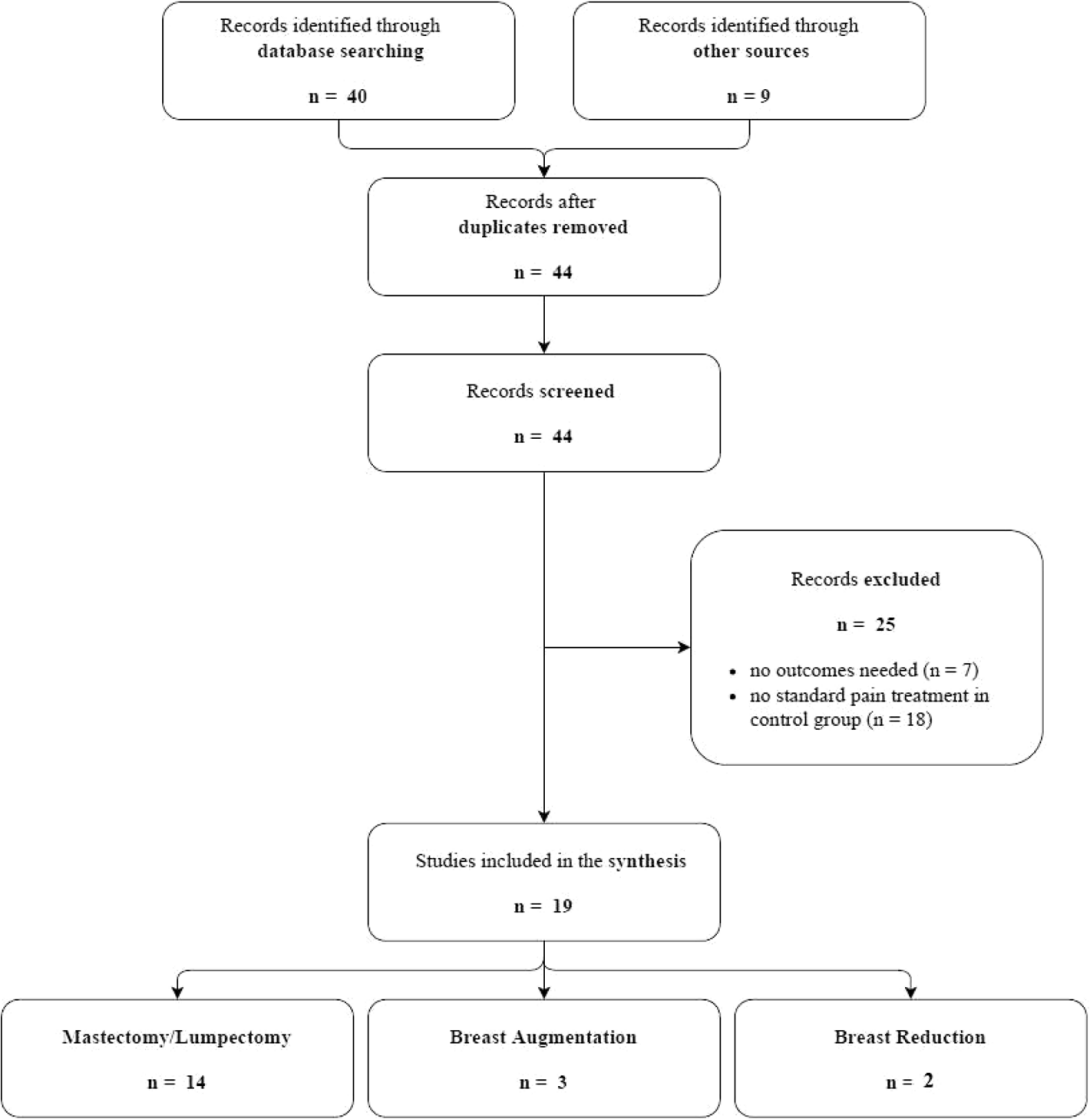
Flow diagram study selection process

Our search strategy identified 40 citations. Searching additional sources yielded an additional 9 potentially eligible citations. Of these, 30 were excluded based on title and abstract screening, because of duplicated papers (*n* = 5), missing outcomes (*n* = 7), or the lack of a standardized pain treatment in the control group (*n* = 18). Thus a total of 19 randomized controlled trials were included in this systematic review. The flow diagram (see Fig. 1) shows the results from the literature search and the study selection process.

The characteristics of included studies and outcomes assessed in this review are presented in Table 2a, b and c.

**Table 2:** Studies characteristics

Author, year	Study	Sites	No.	Type of surgery	Anesthesia	Intervention
**A. LUMPECTOMY/MASTECTOMY**						
***Campbell*** **et al.*,***[10] ***2014***	SB-RCT	New Zeland, 1	79	Lumpectomy/Mastectomy ± ALND	?	Local Infiltration
***Couceiro*** **et al.*,***[11] ***2014***	DB-RCT	Brazil, 1	44	Mastectomy	GA	IV lidocaine
***Cros*** **et al.*,***[12] ***2018***	DB-RCT	France, 1 Canada, 1	127	Lumpectomy/Mastectomy ± SLNB/ALND	GA	Pecs I
***Ferreira Laso*** **et al.*,***[13] ***2014***	DB-RCT	Spain, 1	73	Mastectomy	GA	Infusion pump
***Gürkan*** **et al.*,***[14] ***2018***	SB-RCT	Turkey,?	50	Lumpectomy/Mastectomy ± SLNB/ALND	GA	ESP
***Ilfeld*** **et al.*,***[15] ***2014***	DB-RCT	US,?	60	Mastectomy ± ALND	Sedation	PVB
***Lanier*** **et al .**[16]***2018***	DB-RCT	US,?	45	Mastectomy + reconstruction ± SLNB/ALND	?	Intercostal + pectoral nerve blocks
***Mohamed*** **et al.*,***[17] ***2013***	DB-RCT	Egypt,?	140	Mastectomy ± ALND	GA	Local infiltration
***Neethu*** **et al.*,***[18] ***2018***	RCT	India, 1	60	Mastectomy ± SLNB/ALND	GA	Pecs I - II
***Terkawi*** **et al.*,***[19]***2014***	DB-RCT	US,?	71	Mastectomy	GA	IV lidocaine
***Versyck*** **et al.*,***[20] ***2017***	DB-RCT	Belgium, 1	140	Mastectomy/tumorectomy ±SLNB/ALND	GA	Pecs II
***Wang*** **et al.*,***[21] ***2018***	SB-RCT	China,?	64	Mastectomy + reconstruction ± ALND	GA	Pecs II
***Wang*** **et al.*,***[22] ***2019***	RCT	China,?	61	Mastectomy	GA	Pecs I + SPB
***Yao*** **et al.*,***[23] ***2019***	DB-RCT	China, 1	72	Mastectomy ± ALND	GA	SPB
**B. BREAST AUGMENTATION**						
***Gardiner*** **et al.*,***[24] ***2012***	SB-RCT	Australia,?	40	BA	Sedation	PVB
***Picard*** **et al.*,***[25] ***2017***	SB-RCT	France,?	72	BA	?	Local infiltration
***Schuitemaker*** **et al.*,***[26] ***2019***	DB-RCT	Spain,?	30	BA	GA	Pecs II + SPB
**C. BREAST REDUCTION**						
***Christie*** **et al.*,*** [27] ***2017***	DB-RCT	US, 1	40	BR	GA	Tumescent Anaesthesia
***Valente*** **et al.*,***[28] ***2014***	DB-RCT	Brazil,?	39	BR	GA	Local infiltration

*SB-RCT* Single-Blind Randomized Controlled Trial, *DB-RCT* Double-Blind Randomized Controlled Trial, *SLNB* Sentinel Lymph Node Biopsy, *ALND* Axillary Lymph Node Dissection, *GA* General Anesthesia, *Pecs* Pectoralis nerve block, *ESP* Erector Spinae Plane block, *PVB* ParaVertebral Block, *SPB* Serratus Plane Block, *BA* Breast Augmentation, *BR* Breast Reduction;?, not reported

The surgical procedures performed in the reviewed trials included lumpectomy or mastectomy in 14 of 19 trials [10–23], with additional Sentinel Lymph Node Biopsy (SLNB) or Axillary Lymph Node Dissection (ALND) [10, 12, 14–18, 20, 21, 23]. Three trials included patients undergoing breast augmentation [24–26], and two trials also included patients undergoing breast reduction [27, 28].

The 19 randomized controlled trials involved a total of 1307 patients, of which 749 received nerve blocks, 330 received local infiltration, 115 received IV lidocaine, 73 received infusion of local anesthetic via pump, and 40 received tumescent anesthesia.

According to the COSMIN checklist, all studies included in this review showed an excellent-to-good quality. The majority of clinical trials had a low risk of bias.

### Lumpectomy/mastectomy

In the included studies 1086 patients underwent lumpectomy/mastectomy. There were no demographic differences between the two groups as shown in Table 3.

**Table 3:** Personal and clinical characteristics of patients undergoing lumpectomy/mastectomy

	Patients (***n*** =)		Age (years)		Weight (kg)		Height (cm)		BMI (kg/m^**2**^)	
	Group I	Group C	Group I	Group C	Group I	Group C	Group I	Group C	Group I	Group C
***Campbell*** **et al.*, 2014*** [10]	45	34	59.4	61.7	80.7	73.8	163.3	162.3	30.2	28.1
***Couceiro*** **et al.*, 2014*** [11]	22	22	47.0	52.4	–	–	–	–	28.1	28.2
***Cros*** **et al.*, 2018*** [12]	62	66	60.5	62.0	63.6	65.0	160.0	160.0	24.8	25.6
***Ferreira Laso*** **et al.*, 2014*** [13]	34	39	54.8	57.7	67.2	66.7	–	–	–	–
***Gürkan*** **et al.*, 2018*** [14]	25	25	49.5	49.8	72.4	73.1	161.0	161.0	27.8	28.2
***Ilfeld*** **et al.*, 2014*** [15]	30	30	48.0	49.0	62.0	61.0	165.0	166.0	23.0	24.0
***Lanier*** **et al.*, 2018*** [16]	23	22	48.0	50.0	67.0	70.0	160.0	170.0	26.0	26.0
***Mohamed*** **et al.*, 2013*** [17]	105	35	39.9	38.9	70.2	69.8	160.6	158.5	27.4	27.9
***Neethu*** **et al.*, 2018*** [18]	30	30	50.5	45.6	–	–	–	–	–	–
***Terkawi*** **et al.*, 2014*** [19]	34	37	53.0	54.0	–	–	–	–	26.2	28.2
***Versyck*** **et al.*, 2017*** [20]	45	40	59.6	58.8	67.3	67.0	165.0	165.0	24.7	24.6
***Wang*** **et al.*, 2018*** [21]	30	30	46.8	47.4	–	–	–	–	25.4	24.8
***Wang*** **et al.*, 2019*** [22]	32	29	51.3	55.3	58.7	57.6	162.5	161.0	22.3	22.2
***Yao*** **et al.*, 2019*** [23]	34	34	46.5	47.7	57.2	56.2	160.9	160.8	22.3	21.9
***TOTAL***	**585**	**473**	**51.05 ± 5.6**	**52.16 ± 6.3**	**66.65 ± 6.5**	**66.03 ± 5.8**	**162.04 ± 1.8**	**162.74 ± 3.3**	**25.71 ± 2.3**	**25.83 ± 2.2**

The largest studies involved 140 patients (*Mohamed* et al.*,* [17]*; Versyck* et al.*,* [20]), whereas the smallest consisted of 44 patients (*Couceiro* et al.*,* [11]). All analysed studies were conducted in inpatient settings.

Patients underwent mastectomy, while lumpectomy was performed in 4 studies (*Campbell* et al.*,*[10]*; Cros* et al.*,* [12]*; Gürkan* et al.*,*[14]*; Versyck* et al.*,* [20]). *Lanier* et al [16] and *Wang* et al [21] reported immediate tissue expander or implant based breast reconstruction. Surgical treatment for breast cancer was associated with a sentinel lymph node biopsy (SLNB) or a axillary lymph node dissection (ALND) in 10 papers (*Campbell* et al.*,*[10]*; Cros* et al.*,*[12]*; Gürkan* et al.*,*[14]*; Ilfeld* et al.*,* [15]*; Lanier* et al.*,* [16]*; Mohamed* et al.*,*[17]*; Neethu* et al.*, 2018; Versyck* et al. [20]*;Wang* et al.*, 2018; Yao* et al.*, 2019*).

Regional anesthetic techniques were performed ten times: the most common procedure was pectoral nerve (Pecs) block type I and II (5 studies: *Cros* et al.*,*[12]*; Neethu* et al.*, 2018; Versyck* et al. *2017; Wang* et al.*, 2018; Wang* et al.*, 2019*), followed by serratus plane block (SPB) (2 studies: *Wang* et al.*, 2019; Yao* et al.*, 2019),* erector spinae plane (ESP) block (*Gürkan* et al.*,*[14]), and paravertebral block (PVB) (*Ilfeld* et al.*,*[15]). In *Lanier* et al *2018,* intraoperative nerve blocks of intercostal and pectoral nerves were performed under direct visualization by the attending plastic surgeon at the completion of the mastectomy.

Local infiltration was reported in 3 studies. In *Campbell* et al *2014* [10]*,* patients received 20 mL of bupivacaine 0.25% with or without adrenaline to be infiltrated into the breast wound and a further 20 mL of bupivacianee 0.25% with adrenaline to be infiltrated into the axilla wound when applicable. In *Mohamed* et al *2013,* 5 ml of bupivacaine 0.5% with or without clonidine were diluted with saline 0.9% to 15 mL volume and irrigated into the surgical field before skin closure. An infusion pump of levobupivacaine 0.50% for approximately 48 h was evaluated in *Ferreira Laso* et al *2014.*

*Couceiro* et al [11] and *Terkawi* et al *2014* investigated i.v. lidocaine infusion. In the first paper, a bolus dose of lidocaine was not administered and, after incision, a lidocaine infusion at 3 mg/kg was started. In the other study, lidocaine was administered as a bolus to all patients before anesthetic induction, at a dose of up to 1.5 mg/kg, followed by a lidocaine infusion at 2 mg/kg/h until 2 h after arrival in PACU.

Almost all studies were conducted under general anesthesia, except for *Ilfeld* et al *2014* conducted under sedation. General anesthesia was induced and then maintained with opioids, such as alfentanil (*Ferreira Laso* et al.*, 2014*), fentanyl (*Couceiro* et al.*,* [11]*; Gürkan* et al.*, 2018; Ilfeld* et al.*, 2014; Mohamed* et al.*, 2013; Neethu* et al.*, 2018; Terkawi* et al.*, 2014; Wang* et al.*, 2018*), remifentanil (*Wang* et al.*, 2019*) and sufentanil (*Versyck* et al.*, 2017; Wang* et al.*, 2019; Yao* et al.*, 2019*). For the postoperative pain management, four studies (*Couceiro* et al.*,* [11]*; Cros* et al.*,*[12]*; Ferreira Laso* et al.*, 2014; Ilfeld* et al.*, 2014*) provided infiltration of the chest wall ipsilateral to the mastectomy with local anesthetic; acetaminophen and other NSAIDs were systematically administered. Two studies did not report the anesthesia protocol (*Campbell* et al.*, 2014* [10]*; Lanier* et al.*, 2018*).

#### Pain intensity

Different investigators recorded this outcome on different scales and at different intervals. We normalized all NRS to a zero to 10 range (see Table 4). The majority of authors reported pain intensity at 1, 6, 12, 24 and 48 h after surgery.

**Table 4:** NRS at 1, 6, 12, 24 and 48 h after lumpectomy/mastectomy

	Up to 1 h		Up to 6 h		Up to 12 h		Up to 24 h		Up to 48 h	
	Group I	Group C	Group I	Group C	Group I	Group C	Group I	Group C	Group I	Group C
***Campbell*** **et al.*, 2014*** [10]	–	–	1.75	2	–	–	2.3	1.7	1.8	1.25
***Couceiro*** **et al.*, 2014*** [11]	–	–	–	–	–	–	–	–	–	–
***Cros*** **et al.*, 2018*** [12]	3	3	–	–	–	–	–	–	–	–
***Ferreira Laso*** **et al.*, 2014*** [13]	1.6	6.7	–	–	–	–	0.8	4.2	0.4	3.3
***Gürkan*** **et al.*, 2018*** [14]	2	2	2	2	0	1	0	1	–	–
***Ilfeld*** **et al.*, 2014*** [15]	–	–	–	–	–	–	3.6	3.7	–	–
***Lanier*** **et al.*, 2018*** [16]	3	5	4	5	5	4	4	4	–	–
***Mohamed*** **et al.*, 2013*** [17]	2.67	3.7	2.43	3.6	2.53	3.8	2.43	3.7	2.43	3.8
***Neethu*** **et al.*, 2018*** [18]	1.78	3.08	0.43	0.76	1.20	1.40	0.5	0.53	–	–
***Terkawi*** **et al.*, 2014*** [19]	–	–	2.94	3.88	–	–	2.91	2.66	2.72	3.9
***Versyck*** **et al.*, 2017*** [20]	0.38	0.20	–	–	–	–	–	–	–	–
***Wang*** **et al.*, 2018*** [21]	1.65	4.3	2.45	3.9	1.8	1.9	1	1	–	–
***Wang*** **et al.*, 2019*** [22]	1	3	0.8	4.4	1	2	0.5	1.8	0.2	1.6
***Yao*** **et al.*, 2019*** [23]	1.3	2.7	1.4	2.4	–	–	1.2	1.8	–	–
***TOTAL***	**1.83 ± 0.81**	**3.36 ± 1.65**	**2.02 ± 1.03**	**3.10 ± 1.29**	**1.92 ± 1.57**	**2.35 ± 1.14**	**1.74 ± 1.29**	**2.37 ± 1.27**	**1.51 ± 1.03**	**2.77 ± 1.12**
***p***	0.02*		**0.08**		**0.63**		**0.29**		**0.13**	

**Fig. 2:**
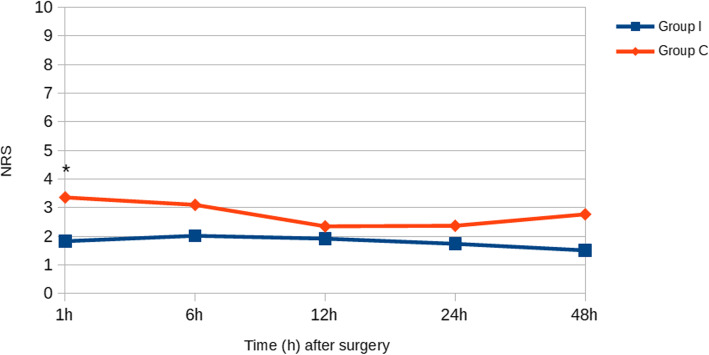
NRS at 1, 6, 12, 24 and 48 h after lumpectomy/mastectomy

As shown in Fig. 2, the average NRS score was 1.83 ± 0.81 at 1 h, 2.02 ± 1.03 at 6 h, 1.92 ± 1.57 at 12 h, 1.74 ± 1.29 at 24 h and 1.51 ± 1.03 at 48 h in the intervention group; in the placebo group NRS scores were recorded of 3.36 ± 1.65 at 1 h, 3.10 ± 1.29 at 6 h, 2.35 ± 1.14 at 12 h, 2.37 ± 1.27 at 24 h and 2.77 ± 1.12 at 48 h.

One study, *Couceiro* et al [11], did not report NRS at any interval: only 2 (9.09%) vs 3 (13.6%) patients in the lidocaine and placebo groups, respectively, experienced severe to very severe pain 24 h after surgery.

#### Opioid consumption

The most frequently used opioids were codeine (*Campbell* et al.*, 2014* [10]*; Couceiro* et al.*,* [11]), fentanyl (*Neethu* et al.*, 2018*), oxycodone (*Campbell* et al.*, 2014*) [10], piritramide (*Versyck* et al. *2017*), tramadol (*Campbell* et al.*, 2014* [10]*; Mohamed* et al.*, 2013; Versyck* et al. *2017*), and sufentanil (*Yao* et al.*, 2019*). Morphine was used in the other studies.

At discharge from PACU, the overall mean amounts of morphine consumed in the intervention and placebo groups were 3.0 ± 3.63 mg and 4.87 ± 5.76 mg, respectively, with the difference being not statistically significant (*p* = 0.51).

After 48 h, the overall mean amounts of morphine consumed in the intervention and placebo groups were, respectively, 2.68 ± 0.88 mg and 4.94 ± 4.61 mg (*p* = 0.18). Among regional anesthetic techniques, postoperative opioid consumption for the first 48 h was respectively 2.14 ± 1.52 mg and 4.84 ± 4.63 mg; no statistically significant difference was observed (*p* = 0.16). The average per patient opioid consumption up to 48 h after surgery was 1.33 ± 1.49 mg vs 3.01 ± 3.05 mg (*p* = 0.52) among patients treated with local infiltration.

Table 5 shows the mean amounts of morphine consumed in the intervention and placebo groups.

**Table 5:** Average per patient opioid consumption in PACU and up to 48 h after lumpectomy/mastectomy

	PACU			Up to 48 h		
	Group I	Group C	***p***	Group I	Group C	***p***
***Campbell*** **et al.*, 2014*** [10]	–	–		3.42	7.33^a^	
***Couceiro*** **et al.*, 2014*** [11]	–	–		–	–	
***Cros*** **et al.*, 2018*** [12]	1.5	3		1.5	3	
***Ferreira Laso*** **et al.*, 2014*** [13]	0	0.7^a^		0	0.7^a^	
***Gürkan*** **et al.*, 2018*** [14]	1	1		5	16^a^	
***Ilfeld*** **et al.*, 2014*** [15]	1	2.4^a^		2.5	5.7	
***Lanier*** **et al.*, 2018*** [16]	8	17		4	5.18	
***Mohamed*** **et al.*, 2013*** [17]	–	–		0.58	1^a^	
***Neethu*** **et al.*, 2018*** [18]	–	–		1.46	2.03^a^	
***Terkawi*** **et al.*, 2014*** [19]	9.35	9.69		11.02	11.61	
***Versyck*** **et al.*, 2017*** [20]	0.18	0.33^a^		0.20	0.37^a^	
***Wang*** **et al.*, 2018*** [21]	–	–		1.75	5.42^a^	
***Wang*** **et al.*, 2019*** [22]	–	–		–	–	
***Yao*** **et al.*, 2019*** [23]	–	–		0.73	1.03^a^	
***TOTAL***	**3.0 ± 3.63**	**4.87 ± 5.76**	**0.51**	**2.68 ± 0.88**	**4.94 ± 4.61**	**0.18**

^a^ Difference statistically significant

In *Couceiro* et al [11]*,* opioid consumption in the first 24 h after surgery was similar in the lidocaine and placebo groups.

#### Adverse events (AEs)

**Fig. 3:**
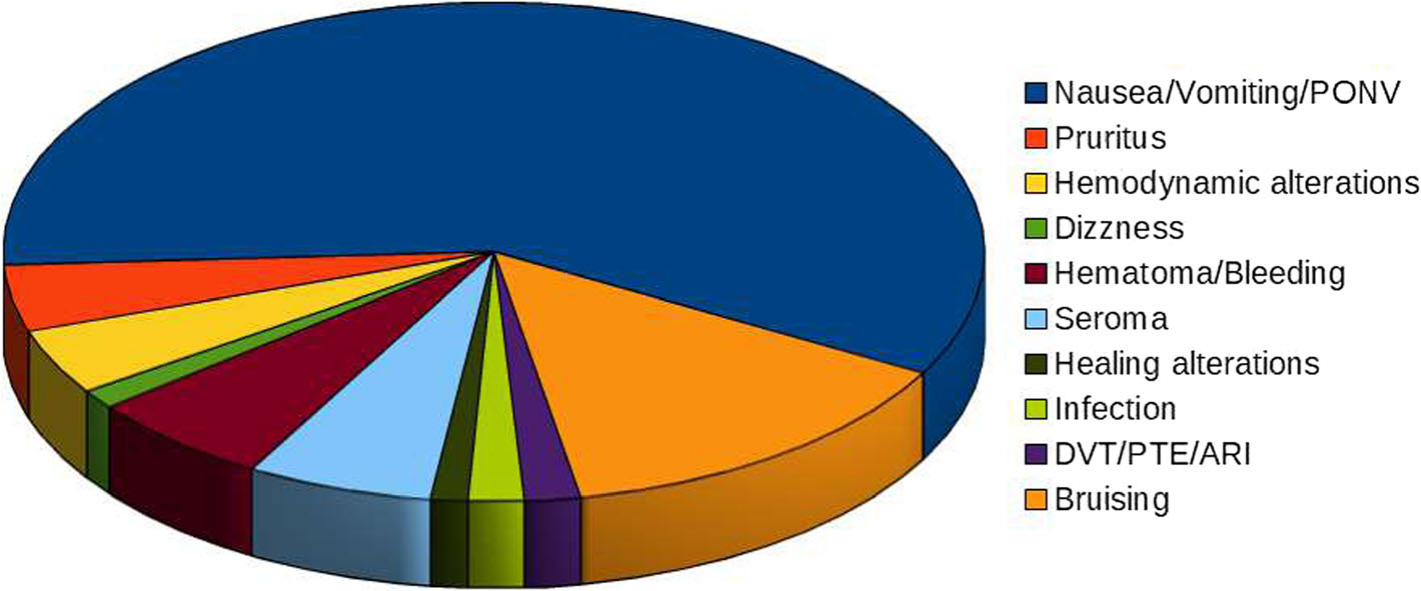
Distribution of adverse events (AEs) among intervention group (lumpectomy/mastectomy)

An adverse event is defined as any undesirable experience associated with the use of a medical product in a patient. A total of 379 AEs were recorded. Three studies (*Couceiro* et al.*,* [11]*; Ilfeld* et al.*, 2014; Versyck* et al.*, 2017)* did not report the number of AEs. The most frequently reported AEs were nausea, vomiting and postoperative nausea and vomiting (PONV), pruritus, dizziness, haematoma/bleeding, seroma and bruising (see Table 6 and Fig. 3). Some studies did not specify the timing of adverse events.

**Table 6:** Number of adverse events (AEs) after lumpectomy/mastectomy

AEs	Group I (*n* =)	Group C (*n* =)	***p***
***Nausea/Vomiting/PONV***	96	139	*0.25*
***Pruritus***	7	16	*0.47*
***Hypotension***	5	2	*0.25*
***Hypertension***	0	1	*0.32*
***Dizzness***	2	15	*0.06*
***Bradycardia***	2	0	*0.32*
***Hematoma/Bleeding***	10	11	*0.85*
***Seroma***	10	11	*0.91*
***Alteration of healing***	2	3	*0.78*
***Infection***	3	2	*0.69*
***DVT***	1	1	*1.00*
***PTE***	1	1	*1.00*
***Acute respiratory infection***	1	1	*1.00*
***Bruising***	23	13	*0.70*
***TOTAL***	**163**	**216**	***0.74***

#### Nausea, vomiting and PONV

Nausea, vomiting and PONV were the most frequent AEs (235 events, 62% of AEs). 96 vs 139 episodes were respectively recorded in intervention vs placebo group (*p* = 0.25). In 9 studies (*Cros* et al.*,*[12]*; Ferreira Laso* et al.*, 2014; Gürkan* et al.*, 2018; Lanier* et al.*, 2018; Neethu* et al.*, 2018; Terkawi* et al.*, 2014; Versyck* et al. *2017; Wang* et al.*, 2018; Yao* et al.*, 2019*) prophylaxis was administered; in the others studies prescription of antiemetic drugs was missed or not reported.

#### Pruritus

A total of 23 cases of pruritus was reported in two studies (*Ferreira Laso* et al.*, 2014; Wang* et al.*, 2019*). No statistically significant difference was observed (7 vs 16, *p* = 0.47).

#### Dizziness

Fifteen episodes were recorded among patients not receiving treatment; only two patients reported dizziness in the intervention group. No statistically significant difference (*p* = 0.06) was observed in these 3 studies (*Ferreira Laso* et al.*, 2014; Wang* et al.*, 2019; Yao* et al.*, 2019*).

#### Haematoma/bleeding

Three studies (*Campbell* et al.*, 2014* [10]*; Cros* et al.*,*[12]*; Ferreira Laso* et al.) reported a total of 21 episodes (10 vs 11, *p* = 0.85).

#### Seroma

Seroma was found in 10 cases in the intervention group vs 11 cases in the placebo group (*Campbell* et al.*, 2014* [10]*; Ferreira Laso* et al.*, 2014*) with no statistically significant difference (*p* = 0.91).

#### Bruising

*Campbell* et al *2014* [10] reported 36 episodes of bruising (20 vs 16, *p* = 0.70).

#### Others

***Haemodynamic*** changes were rarely reported. Hypotension was reported in 3 studies (*Cros* et al.*,* [12]*; Ferreira Laso* et al.*, 2014; Mohamed* et al.*, 2013*) for a total of 7 AEs (5 vs 2, *p = 0.25*). *Ferreira Laso* et al *2014* reported a case of hypertension. Two episodes of bradycardia were reported from *Mohamed* et al *2013.*

Infection was observed, respectively, 3 vs 2 times in intervention and placebo groups in two studies (*Campbell* et al.*, 2014* [10]*; Ferreira Laso* et al.); no statistically significant difference was noticed (*p* = 0.69). Deep vein thrombosis (DVT), pulmonary thromboembolism (PTE) and acute respiratory infection were equally distributed (1 vs 1, *p* = 1.0) in *Ferreira Laso* et al *2014.*

#### Patient satisfaction

Patient satisfaction results were presented as different degrees of subjective satisfaction levels. We normalized all of them to “satisfied / not satisfied”.

**Fig. 4:**
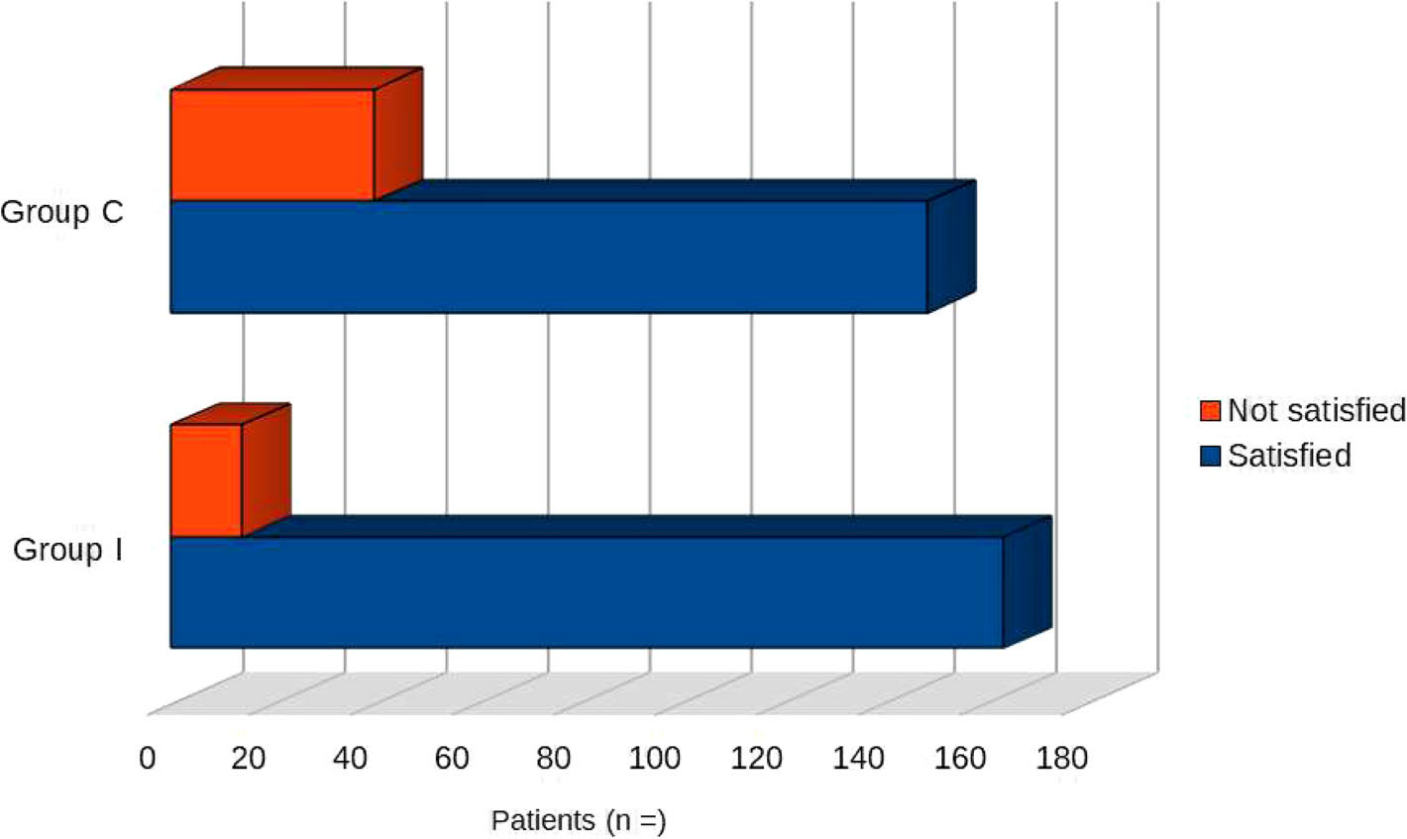
Patient satisfaction after lumpectomy/mastectomy

Only 5 studies (*Cros* et al.*,*[12]*; Ferreira Laso* et al.*, 2014; Lanier* et al.*, 2018; Neethu* et al.*, 2018; Wang* et al.*, 2019;*) were available for analysis of satisfaction (see Table 7 and Fig. 4).

**Table 7:** Patient satisfaction after lumpectomy/mastectomy

	Group I		Group C	
	Satisfied	Not satisfied	Satisfied	Not satisfied
***Campbell*** **et al.*, 2014*** [10]	*–*	*–*	*–*	*–*
***Couceiro*** **et al.*, 201 4***[11]	*–*	*–*	*–*	*–*
***Cros*** **et al.*, 2018*** [12]	*61*	*1*	*64*	*1*
***Ferreira Laso*** **et al.*, 2014*** [13]	*32*	*2*	*37*	*2*
***Gürkan*** **et al.*, 2018*** [14]	*–*	*–*	*–*	*–*
***Ilfeld*** **et al.*, 2014*** [15]	–	–	–	–
***Lanier*** **et al.*, 2018*** [16]	*23*	0	23	0
***Mohamed*** **et al.*, 2013*** [17]	*–*	*–*	*–*	*–*
***Neethu*** **et al.*, 2018*** [18]	*25*	*5*	*10*	*20*
***Terkawi*** **et al.*, 2014*** [19]	*–*	*–*	*–*	*–*
***Versyck*** **et al.*, 2017***^***a***^ [20]	–	*–*	*–*	*–*
***Wang*** **et al.*, 2018*** [21]	*–*	*–*	*–*	*–*
***Wang*** **et al.*, 2019*** [22]	*23*	*6*	*15*	*17*
***Yao*** **et al.*, 2019***^***b***^ [23]	–	–	–	–
***TOTAL***	***164***	***14***	***149***	***40***

^a^ Both patient-groups were very satisfied about their management (9.6 ± 0.6 and 9.1 ± 1.8 on a 10-point scale, *p* = 0.21)

^b^Patient satisfaction scores were higher in the SPB group

Among the intervention group, there were 164 satisfied patients vs 14 not satisfied patients; in the placebo group 149 patients were satisfied and 40 not satisfied. No statistically significant difference was observed between the two groups (satisfied, *p* = 0.28; not satisfied, *p* = 0.14).

In *Versyck* et al *2017,* both patient-groups were very satisfied with their management; while in *Yao* et al *2019,* patient satisfaction scores were higher in the SPB group.

### Breast augmentation

One hundred forty-two patients underwent breast augmentation. As shown in Table 8, the groups were similar in age, weight, height and body mass index.

**Table 8:** Personal and clinical characteristics of patients undergoing breast augmentation

	Patients (***n*** =)		Age (years)		Weight (kg)		Height (cm)		BMI (kg/m^**2**^)	
	Group I	Group C	Group I	Group C	Group I	Group C	Group I	Group C	Group I	Group C
***Gardiner*** **et al.*, 2012*** [24]	20	20	33.4	34.9	–	–	–	–	20.5	20.1
***Picard*** **et al.*, 2017*** [25]	29	43	32.4	34.6	–	–	–	–	–	–
***Schuitemaker*** **et al.*, 2019*** [26]	15	15	33.0	33.0	52.0	54.0	163.0	163.0	20.0	20.0
***TOTAL***	**64**	**78**	**32.93 ± 0.41**	**34.16 ± 0.83**	**52.0**	**54.0**	**163.0**	**163.0**	**20.25 ± 0.25**	**20.05 ± 20.04**

All patients underwent subpectoral bilateral cosmetic breast augmentation.

Regional anesthetic techniques were performed in two studies: PVB in *Gardiner* et al [24]*;* association of Pecs type II and PSB in *Schuitemaker* et al [26]*.* In the other study, *Picard* et al [25]*,* a local infiltration was performed.

Patients received general anesthesia in *Schuitemaker* et al [26]*,* and sedation in *Gardiner* et al [24]*.* In both studies patients received fentanyl. *Picard* et al [25] did not report the anesthesia protocol.

#### Pain intensity

Different investigators recorded this outcome on different scales and at different intervals. We normalized all NRS to a zero to 10 range (see Table 9). The majority of authors reported pain intensity at 1, 6, 24 and 72 h after surgery.

**Table 9:** NRS at 1, 6, 24 and 72 h after breast augmentation

	Up to 1 h		Up to 6 h		Up to 24 h		Up to 72 h	
	Group I	Group C	Group I	Group C	Group I	Group C	Group I	Group C
***Gardiner*** **et al.*, 2012*** [24]	3.9	5.2	–	–	–	–	3.3	4.7
***Picard*** **et al.*, 2017*** [25]	–	–	–	–	4.8	5.1	2.8	3.7
***Schuitemaker*** **et al.*, 2019*** [26]	2.9	5.3	3.0	3.0	2.5	3.0	–	–
***TOTAL***	**3.4 ± 0.5**	**5.25 ± 0.05**	**3.0**	**3.0**	**3.65 ± 1.15**	**4.05 ± 1.05**	**3.05 ± 0.25**	**4.2 ± 0.5**
***p***	**0.06**		**–**		**0.82**		**0.17**	

**Fig. 5:**
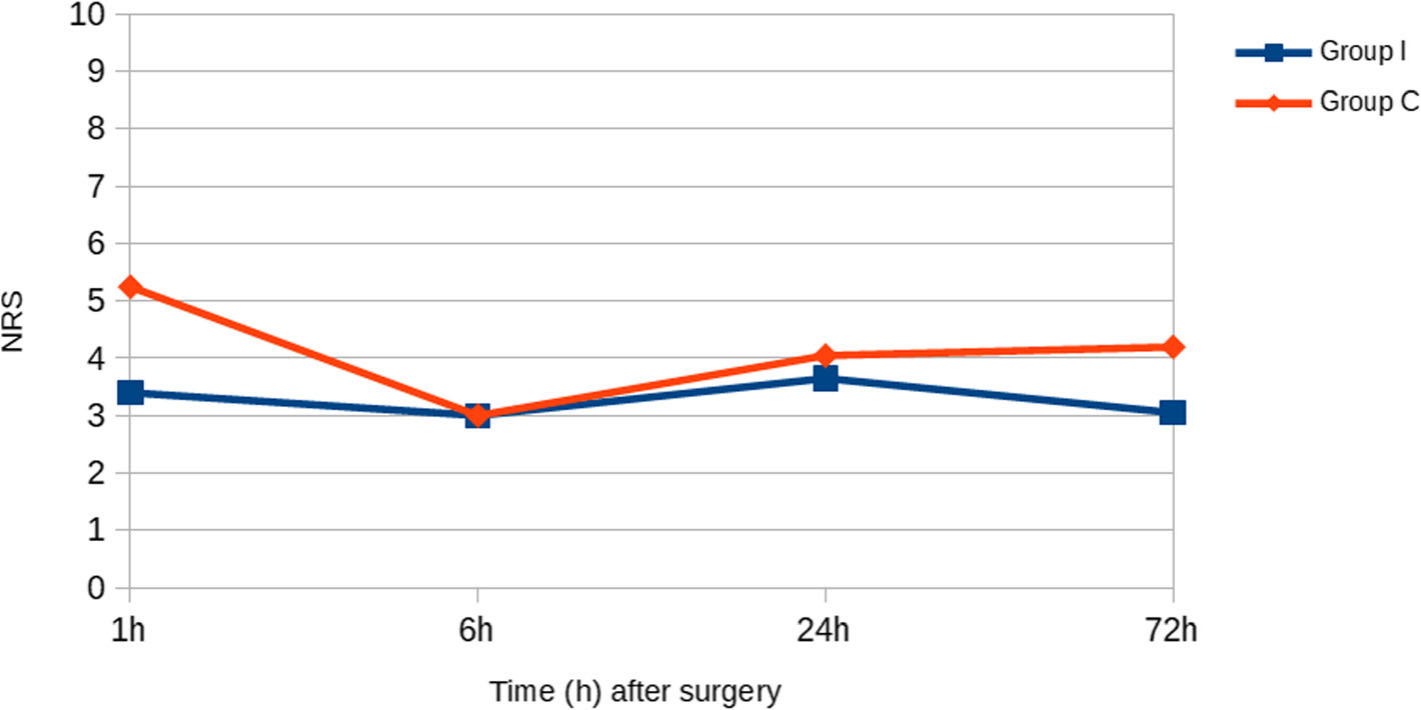
NRS at 1, 6, 24 and 72 h after breast augmentation

As shown in Fig. 5, the average NRS scores were 3.4 ± 0.5 at 1 h, 3.0 at 6 h, 3.65 ± 1.15 at 24 h and 3.05 ± 0.25 at 72 h in the intervention group; in the placebo group NRS scores were recorded of 5.25 ± 0.05 at 1 h, 3.0 at 6 h, 3.65 ± 1.15 at 24 h and 4.2 ± 0.5 at 72 h.

#### Opioid consumption

In *Gardiner* et al [24]*,* 6 patients in the placebo group required supplementary opioid use. No patient received opioids in the intervention group.

After 24 h in *Schuitemaker* et al [26], the overall mean amounts of morphine consumed in the intervention and placebo groups were 0.2 ± 0.8 mg and 0.6 ± 1.2 mg, respectively, with the difference being not statistically significant (*p* = 0.29).

No data on opioid consumption was available in *Picard* et al [25]*.*

#### Adverse events (AEs)

A total of 19 (9 vs 10) AEs were recorded.

*Gardiner* et al [24] reported 12 episodes of nausea and vomiting (5 vs 7, *p* = 0.36). In this study, hypotension occurred 3 times among patients in the intervention group and once in the placebo group. A single case of bradycardia occurred in both groups.

No differences were observed between groups concerning the appearance of AEs in *Schuitemaker* et al [26]*.*

No adverse effects were reported in *Picard* et al *2017.*

#### Patient satisfaction

Only *Schuitemaker* et al [26] reported data about patient satisfaction: after 24 h, 80% vs 53% of patients in intervention and placebo groups were satisfied.

### Breast reduction

Seventy-nine patients underwent breast reduction. The mean age was 38.28 ± 2.71 years vs 38.78 ± 3.21 years (see Table 10).

**Table 10:** Personal and clinical characteristics of patients undergoing breast reduction

	***Patients, n***		***Age, years***		***Weight, kg***		***Height, cm***		***BMI, kg/m***^***2***^	
	***Group I***	***Group C***	***Group I***	***Group C***	***Group I***	***Group C***	***Group I***	***Group C***	***Group I***	***Group C***
***Christie*** **et al.*, 2017*** [27]	20	20	41.0	42.0	–	–	–	–	33.0	31.0
***Valente*** **et al.*, 2014*** [28]	18	20	35.57	35.57	–	–	–	–	–	–
***TOTAL***	**38**	**40**	**38.28 ± 2.71**	**38.78 ± 3.21**	**–**	**–**	**–**	**–**	**33.0**	**31.0**

All patients underwent breast reduction surgery.

Tumescent anesthesia was performed in *Christie* et al *2017;* in *Valente* et al *2014* [28]*,* patients received local infiltration. All patients in these studies underwent general anesthesia.

#### Pain intensity

Different investigators recorded this outcome on different scales and at different intervals. We normalized all NRS to a zero to 10 range (see Table 11). The authors reported pain intensity at 24 h after surgery.

**Table 11:** NRS at 24 h after breast reduction

	Up to 24 h	
	Group I	Group C
***Christie*** **et al.*, 2017*** [27]	4.28	4.00
***Valente*** **et al.*, 2014*** [28]	0.83	1.71
***TOTAL***	**2.55 ± 1.72**	**2.85 ± 1.14**
***p***	**0.89**	

The average NRS score was 2.55 ± 1.72 in the intervention group; in the placebo group a NRS score was recorded of 2.85 ± 1.14 (*p* = 0.89).

#### Opioid consumption

After 24 h in *Christie* et al *2017*, the overall mean amounts of morphine consumed in the intervention and placebo groups were 0.58 mg and 0.64 mg, respectively, with the difference being not statistically significant (*p* = 0.71).

No data on opioid consumption is available in *Valente* et al *2014* [28]*.*

#### Adverse events (AEs)

There was no significant difference in occurrence of nausea or vomiting in the first 24 h between the two groups in *Christie* et al *2017*. No adverse effects were reported in *Valente* et al *2014* [28]*.*

#### Patient satisfaction

The level of satisfaction was not reported in these studies.

## Discussion

Our systematic review is the first to demonstrate the analgesic benefits of locoregional anesthesia following breast surgery, and to demonstrate the clinical utility of these techniques. For perioperative and postoperative analgesia, locoregional anaesthesia can be used as a standalone anaesthetic technique, or in association with sedation or general anaesthesia. In almost all the studies of this review, locoregional anesthesia was performed to reduce pain as an “adjuvant technique”.

### Lumpectomy/mastectomy

#### Pain intensity

Pain intensity on a numeric rating scale (NRS) was lower in the intervention group than in the placebo group at 1, 6, 12, 24 and 48 h after surgery. The difference of NRS at 1 h between the groups was statistically significant (*p* = 0.02); no statically significant difference was reported at other intervals.

In the first hour after surgery, all patients in the intervention group reported NRS lower than 4; instead in the placebo group, two studies (*Ferreira Laso* et al.*, 2014; Wang* et al.*, 2018*) experienced, respectively, a mean pain intensity of 6.7 and 4.3. In the placebo group we also found NRS higher than 4 in *Wang* et al *2019* after 6 h and in *Ferreira Laso* et al *2014* after 24 h.

We considered NRS lower than 4 as an optimal cut-off point between mild and moderate pain. This cut-off was identified as the tolerable pain threshold [29].

#### Opioid consumption

Postoperative use of opioids was lower in the interventional group both upon discharge from the PACU and after 48 h, although there was no statistically significant difference. The difference in opioid use was statistically significant in three studies (*Ferreira Laso* et al.*, 2014; Ilfeld* et al.*, 2014; Versyck* et al. *2017*) at the time of discharge from the PACU. Eight studies (*Campbell* et al.*, 2014* [10]*; Ferreira Laso* et al.*, 2014; Gürkan* et al.*, 2018; Mohamed* et al.*, 2013; Neethu* et al.*, 2018; Versyck* et al. *2017; Wang* et al.*, 2018; Yao* et al.*, 2019*) reached statistical significance after 48 h from surgery.

#### Safety

No statistically significant difference was noticed (*p* = 0.74) between interventional and placebo groups. Among the intervention group a total of 163 AEs was reported. Nausea, vomiting or PONV were the most common (59%), followed by bruising (14%), haematoma/bleeding (6%), seroma (6%), pruritus (4%) and haemodynamic alterations (4%), such as hypotension, hypertension or bradycardia.

#### Patient satisfaction

Patient satisfaction was high, with minimum 92% of satisfaction among patients treated with locoregional anesthesia. The satisfaction rate was also high in the placebo group (79%).

### Breast augmentation

Pain intensity on a numeric rating scale (NRS) was lower in the intervention group than in the placebo group at 1, 6, 24 and 72 h after surgery. No statically significant difference was reported at these intervals.

Postoperative use of opioids was lower in the interventional group after 24 h, although there was no statistically significant difference.

Concerning safety, no difference was noticed between interventional and placebo groups.

### Breast reduction

Pain intensity on a numeric rating scale (NRS) was lower in the intervention group than in the placebo group after 24 h. No statically significant difference was reported.

Postoperative use of opioids was lower in the interventional group after 24 h, although there was no statistically significant difference.

No difference was noticed between interventional and placebo groups about AEs incidence.

General anesthesia is the conventional, most frequently used anesthetic technique. Various locoregional anesthetic techniques are also used for breast surgeries. These include local wound infiltration [30], tumescent anesthesia [31], regional anesthetic techniques, such as pectoral nerve (Pecs) blocks type 1 and 2 [32, 33], serratus plane block (SPB) [34], and parasternal block (PSB) [35], pain pump [36, 37], and intravenous regional block [7, 38].

We considered continuous IV infusion of lidocaine for our review. Various are the reasons that led us to consider this technique. Local anesthetics exert their pharmacological action through the block of sodium channels in neural tissues, thereby interrupting neuronal transmission. This action is best demonstrated when the drug comes directly in contact of neural tissues. However, the systemic effects of lidocaine are also probably or at least partially, related to this mechanism [39]. The IV lidocaine shares many of the effects of local anesthetics when used in regional anesthesia techniques. It can lead to better postoperative analgesia, reduced opioid consumption and improved intestinal motility [40]. In addition to the analgesic action, local anesthetics have anti-inflammatory action, justifying also the use of intravenous lidocaine to modulate the inflammatory response resulting from postoperative pain [41].

Locoregional anesthesia provides effective anesthesia and analgesia in the perioperative setting. The beneficial analgesic effect of the regional block is well known, and also confirmed in our analysis. After mastectomy, the use of locoregional anesthesia techniques seems to reduce pain especially in the first hour after the end of the surgery.

Other potentially beneficial effects of locoregional anaesthesia and analgesia on other perioperative outcomes include decreased need for opioids for controlling postoperative pain, decreased postoperative nausea and vomiting, fewer complications and increased patient satisfaction. In our review, there was no statistically significant difference between the analysed anesthesia techniques.

The effective management and relief of postoperative pain plays a vital role in overall surgical outcome. Untreated pain has been linked to prolonged hospital stays, deep venous thrombosis, pulmonary embolism, pneumonia, bowel dysmotility, insomnia, and impaired wound healing [42]. Reduced occurrence of nausea and vomiting is related to better analgesia and opioids/inhalational anaesthetics sparing effect by regional blocks [43, 44]. All this improves post-operative recovery and shortens hospitalization stay.

#### Limitations

Our review has several limitations. First, some outcomes were characterized by high levels of heterogeneity. Reasons for this may be attributable to subtle variations in surgical technique and differences in anesthetic and analgesic regimens. Second, for 3 studies included in this review (*Campbell* et al *2014* [10], *Lanier* et al *2018* and *Picard* et al [25]*),* it is not possible to assess whether only regional anesthesia for breast surgery was performed. The impact of locoregional anesthesia on nociception as a “pure” or “adjuvant” technique is different, notably because of the different dosage of local anesthetics. We decided not to exclude these studies and to accept the possible bias during the analysis. Third, many of the included studies had small sample sizes, which decreases their effect and limits external validity. Fourth, another major limitation of this review was the large and unexplained statistical heterogeneity between the studies. Finally, we included two studies (*Couceiro* et al [11]*; Terkawi* et al *2014*) analysing the use of i.v. lidocaine. Systemic lidocaine is not “really” a locoregional anesthesia technique, nevertheless we decided to include it in our review accepting the possible bias arising from systemic effects of this local anesthetic.

All these limitations reduced the quality of the evidence for most of the outcomes.

## Conclusion

In this systematic review we found evidence for an effect of locoregional anesthesia on the pain due to breast surgery as one the major predefined outcomes. The difference of NRS at 1 h between the groups was statistically significant among patients who underwent lumpectomy/mastectomy; no statically significant difference was reported at other intervals.

The postoperative opioids consumption, the incidence of PONV and other AEs, and the patient satisfaction were not different among patients who underwent locoregional anesthesia or conventional analgesia.

## Data Availability

Dataset derived from public resources and are stored in our computer system at the anesthesia institute of the University Hospital “Luigi Vanvitelli” (Naples – Italy). Datasets are available from the corresponding author on reasonable request.

## Abbreviations

AEs: Adverse Events
ALND: Axillary Lymph Node Dissection
BMI: Body Mass Index
DVT: Deep Vein Thrombosis
ESP: Erector Spinae Plane
IV: Intravenous
NRS: Numeric Rating Scale
PACU: Post-Anesthesia Care Unit
Pecs: Pectoralis nerve
PONV: Postoperative Nausea and Vomiting
PTE: Pulmonary Thromboembolism
PVB: Paravertebral Block
SLNB: Sentinel Lymph Node Biopsy
SPB: Serratus Plane Block
